# Dyspnoea and chest pain as the presenting symptoms of pneumomediastinum: two cases and a review of the literature

**DOI:** 10.5830/CVJA-2015-035

**Published:** 2015

**Authors:** Hasan Kara, Hasan Gazi Uyar, Degirmenci Selim, Bayir Aysegul, Ahmet Ak, Murat Oncel

**Affiliations:** Department of Emergency Medicine, Faculty of Medicine, Selçuk University, Konya, Turkey; Department of Emergency Medicine, Faculty of Medicine, Selçuk University, Konya, Turkey; Department of Emergency Medicine, Faculty of Medicine, Selçuk University, Konya, Turkey; Department of Emergency Medicine, Faculty of Medicine, Selçuk University, Konya, Turkey; Department of Emergency Medicine, Faculty of Medicine, Selçuk University, Konya, Turkey; Department of Thoracic Surgery, Faculty of Medicine, Selcuk University, Konya, Turkey

**Keywords:** trauma, spontaneous, mediastinum, emergency department

## Abstract

Pneumomediastinum is the presence of air in the mediastinum. It may occur as spontaneous, traumatic, or iatrogenic pneumomediastinum. Although spontaneous pneumomediastinum is usually observed in healthy young men, traumatic pneumomediastinum may be caused by blunt or penetrating trauma to the chest and neck. Pneumomediastinum is a clinical condition with potential complications that cause high morbidity and mortality rates. Pneumomediastinum also may develop without tracheal or oesophageal injury after spontaneous or blunt chest, neck and facial injuries, and it may be accompanied by pneumothorax.

We treated two patients who had pneumomediastinum. Case 1 was a 20-year-old man who had pain and dyspnoea around the sternum for one hour, as a result of a blow from an elbow during a football match. Case 2 was a 23-year-old man who had a two-day history of dyspnoea and chest pain with no history of trauma. In both patients, diagnosis of pneumomediastinum was confirmed with thoracic computed tomography scans, and the condition resolved within five days of in-patient observation. In conclusion, the diagnosis of pneumomediastinum should be considered for all patients who present to the emergency department with chest pain and dyspnoea.

## Abstract

Pneumomediastinum, also known as mediastinal emphysema, is the presence of air or other gas in the mediastinum.^1^ Pneumomediastinum can be categorised as traumatic, spontaneous, or iatrogenic, and it also may be categorised as spontaneous or secondary. Spontaneous pneumomediastinum may occur in situations that increase alveolar pressure, such as coughing, vomiting, straining, or Valsalva manoeuvre, which may cause spontaneous rupture of the alveoli. These conditions may occur with asthma, chronic obstructive pulmonary disease, diabetic keto-acidosis, excessive exercise, cannabis or cocaine intake, and diffuse interstitial fibrosis. In addition, severe coughing that may cause mediastinal emphysema may occur with pertussis, diphtheria, influenza, bronchiolitis, or acute bronchitis in children.

Iatrogenic pneumomediastinum may develop after tracheostomy induced by barotrauma during mechanical ventilation or as a result of rupture of the tracheo-bronchial tree or oesophagus during endoscopy. Traumatic pneumomediastinum may occur as a result of blunt or penetrating chest, head or neck, or eye injuries.^2^,^3^

Traumatic and spontaneous pneumomediastinum have similar symptoms, most commonly retrosternal chest pain that begins acutely. In addition, common symptoms and signs include neck pain, neck swelling, dyspnoea, cough, nasal voice, dysphagia, anxiety, increased salivation, hoarseness and fever.

The clinical presentation is variable and may range from vague symptoms to life-threatening respiratory failure. The patient may have subcutaneous emphysema present in the neck and chest, a Hamman sign with heart auscultation (crackling sounds synchronous with the heartbeat), or cardiovascular collapse.^4^

The purpose of this study was to report the experience with two patients who had isolated pneumomediastinum that presented with dyspnoea and chest pain.

## Case reports

## Case 1

A 20-year-old man had pain and dyspnoea around the sternum for one hour as a result of a blow from an elbow during a football match, and he was admitted to the emergency department. His past medical history was non-contributory. The blood pressure was 130/75 mmHg, pulse was 87 beats per minute, respiratory rate was 16 breaths per minute, temperature was 36.9°C, and transcutaneous oxygen saturation was 96% on room air. He had crepitus on palpation around the sternal notch. Auscultation of the heart revealed a loud crunch-like sound during systole consistent with Hamman sign. Neurological and abdominal examinations showed no abnormalities.

**Fig. 1.**
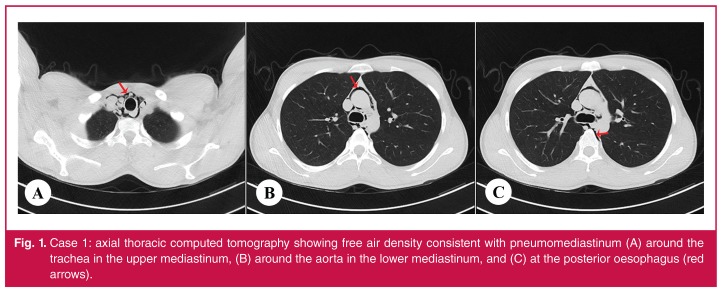
Case 1: axial thoracic computed tomography showing free air density consistent with pneumomediastinum (A) around the trachea in the upper mediastinum, (B) around the aorta in the lower mediastinum, and (C) at the posterior oesophagus (red arrows).

Laboratory tests, including cardiac enzymes and an electrocardiogram, were normal. A postero-anterior chest radiograph was normal, but a chest computed tomography (CT) scan showed subcutaneous emphysema and pneumomediastinum (Fig. 1). There was no evidence of pneumothorax, pneumopericardium, pulmonary parenchymal injury, rib fractures, or tracheal or bronchial injuries.

The patient was transferred to the thoracic surgery department and admitted to hospital for observation and non-surgical treatment. His progress was uneventful and he was discharged after four days. Written informed consent was obtained from the patient for the publication of this case report.

## Case 2

A 23-year-old man was admitted to the emergency department because of a two-day history of dyspnoea and chest pain. He had no history of trauma. The blood pressure was 120/85 mmHg, pulse was 91 beats per minute, respiratory rate was 18 breaths per minute, temperature was 37°C, and transcutaneous oxygen saturation was 93% on room air. There was tenderness to palpation in the right hemithorax and around the sternum. The breath sounds were normal and equal in both lungs.

**Fig. 2.**
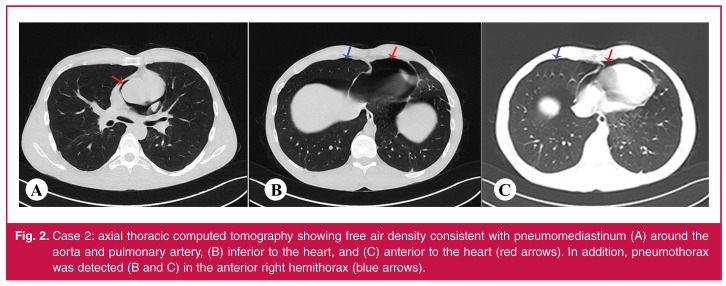
Case 2: axial thoracic computed tomography showing free air density consistent with pneumomediastinum (A) around the aorta and pulmonary artery, (B) inferior to the heart, and (C) anterior to the heart (red arrows). In addition, pneumothorax was detected (B and C) in the anterior right hemithorax (blue arrows).

Laboratory tests, including cardiac enzymes and an electrocardiogram, were normal. The postero-anterior chest radiograph showed a right pneumothorax and transparency that was consistent with left mediastinal air. Thoracic CT scan showed right pneumothorax and pneumomediastinum (Fig. 2).

The patient was transferred to the thoracic surgery department and admitted to hospital for observation and non-surgical treatment. His progress was uneventful and he was discharged after five days. Written informed consent was obtained from the patient for the publication of this case report.

## Discussion

The chief complaint on presentation to the emergency department in both patients included chest pain and dyspnoea. The first patient had traumatic pneumomediastinum as a result of blunt chest trauma, which is a rare clinical condition. The second patient had spontaneous pneumomediastinum. In this study, we investigated the diagnosis and treatment of the two patients who had pneumomediastinum with similar clinical symptoms but different causes.

Pneumomediastinum may have varied causes, including tracheobronchial or oesophageal rupture that may cause an air leak into the mediastinum.^5^ Pneumomediastinum is a potentially ominous sign because it may have severe complications. The differential diagnosis of chest pain, dyspnoea and subcutaneous emphysema may include acute pulmonary and cardiac conditions, such as pericarditis, pulmonary embolism, pneumonia and pneumothorax, and oesophageal perforation, spasm and reflux disease. These potential causes may be less likely in patients who have traumatic or spontaneous pneumomediastinum.^4^

Spontaneous pneumomediastinum is a rare clinical condition that typically is observed in young men, and symptoms usually resolve spontaneously after diagnosis.^6^,^7^ Traumatic pneumomediastinum may be accompanied by subcutaneous emphysema, pneumothorax, rib fractures and pneumopericardium. Iatrogenic pneumomediastinum may develop as a result of bronchial or oesophageal rupture during endoscopy, barotrauma during mechanical ventilation, or after tracheostomy.^8^

The clinical course of pneumomediastinum is variable. Patients may have mild complaints or life-threatening respiratory distress. Patients usually present with chest pain localised to the sternum. They also may have dysphagia, hoarseness, a foreign body sensation in the throat, and dyspnoea. Subcutaneous emphysema detected on physical examination may occur as a result of the spread of extra-alveolar air to the neck, face and anterior chest wall. In addition to subcutaneous emphysema, physical examination may show a crackling sound synchronous with the heartbeat (Hamman sign), which is pathognomonic for pneumomediastinum.^8^

Although the reasons for hospital admission were the same in both patients (chest pain and dyspnoea), the fact that blunt chest trauma was accompanied by subcutaneous emphysema in the first case was an important finding in the diagnosis of pneumomediastinum. Patients who are suspected of having pneumomediastinum should be evaluated with postero-anterior and lateral chest radiography that includes the cervical area. Although CT scan is more sensitive than ordinary chest radiography in detecting pneumomediastinum, the diagnosis is often verified with a careful history and chest radiographs.

Radiographs may show a vertical lucent line on the left side of the heart and aortic arch, lucent line through the retrosternal, pericardiac and paratracheal areas, or subcutaneous emphysema of the shoulders and neck.^9^-^11^ Suggestive radiographic signs may include the thymic sail sign (appearance of thymus as a triangular sail), ring-around-the-artery sign (lucency along the right pulmonary artery on the lateral radiograph caused by mediastinal air), tubular artery sign (air outlining the major aortic branches), double bronchial wall sign (air outlining the bronchial wall), continuous diaphragm sign (lucency above the diaphragm), and extrapleural sign (pulmonary opacity with oblique margins). The CT scan should be reserved for evaluation of underlying lung disease or other accompanying conditions.

In the present study, postero-anterior chest radiography was normal in the first patient, but the second patient had a right apical pneumothorax and left hyperlucency, with the appearance of a linear band that suggested the presence of mediastinal air. Chest radiography may be normal in 30% of patients. Therefore, the most sensitive method, thoracic CT scan, could be useful in diagnosing pneumomediastinum when there is clinical suspicion but non-contributory radiographs. In addition, bronchoscopy and oesophagoscopy can be considered because they may show possible ruptures in the bronchial tree and oesophagus (Boerhaave syndrome); in such cases, surgical intervention should be considered. In some cases, contrast studies and mediastinoscopy may be helpful.

In the treatment of pneumomediastinum, supportive care should be considered when there is no bronchial injury, oesophageal injury, or bullous structure from lung disease that may cause air leakage.^11^ The treatment of pneumomediastinum in the emergency department includes airway and haemodynamic stabilisation, and treatment to prevent further complications such as tension pneumomediastinum and mediastinitis. Patients who have pneumomediastinum should be observed and provided with supplemental oxygen. Treatment should be non-surgical until symptoms disappear within four to five days.^12^,^13^

Both of our patients received supportive treatment in the thoracic surgery department and were followed with daily postero-anterior chest radiography. They were discharged on hospital day four and five, respectively, without complications.

## Conclusion

Pneumomediastinum is a clinical condition that can vary from a mild to life-threatening clinical situation. This diagnosis should be considered for all patients who present to the emergency department with chest pain and dyspnoea. Pneumomediastinum also may develop spontaneously or after blunt chest, neck, facial, or eye injury, with or without tracheal or oesophageal injury. Despite normal chest radiographs, patients suspected of having traumatic or spontaneous pneumomediastinum should have a CT scan. Patients who have pneumomediastinum should be hospitalised for observation because the condition may be associated with complications, including death.

## References

[1] Kikuchi N, Ishii Y, Satoh H, Ohtsuka M, Hizawa N, Ohta Y (2008). Spontaneous pneumomediastinum after air travel.. Am J Emerg Med.

[2] Maravelli AJ, Skiendzielewski JJ, Snover W (2000). Pneumomediastinum acquired by glass blowing.. J Emerg Med.

[3] Özhasenekler A, Gökhan Ş, Yilmaz F, Tan Ö, Nasir A (2010). Pneumomediastinum and pneumothorax after blunt neck trauma [in Turkish].. J Acad Emerg Med Case Reports.

[4] Caceres M, Ali SZ, Braud R, Weiman D, Garrett HE Jr (2008). Spontaneous pneumomediastinum: a comparative study and review of the literature.. Ann Thorac Surg.

[5] Wintermark M, Schnyder P (2001). The Macklin effect: a frequent etiology for pneumomediastinum in severe blunt chest trauma.. Chest.

[6] De Luca G, Petteruti F, Tanga M, Luciano A, Lerro A (2011). Pneumomediastinum and subcutaneous emphysema unusual complications of blunt facial trauma.. Indian J Surg.

[7] Esayag Y, Furer V, Izbicki G (2008). Spontaneous pneumomediastinum: is a chest X-ray enough? A single-center case series.. Isr Med Assoc J.

[8] Vanzo V, Bugin S, Snijders D, Bottecchia L, Storer V, Barbato A (2013). Pneumomediastinum and pneumopericardium in an 11-year-old rugby player: a case report.. J Athl Train.

[9] Pekcan S, Gokturk B, Uygun Kucukapan H, Arslan U, Fındık D (2014). Spontaneous pneumomediastinum as a complication in human bocavirus infection.. Pediatr Int.

[10] Mansella G, Bingisser R, Nickel CH (2014). Pneumomediastinum in blunt chest trauma: a case report and review of the literature.. Case Rep Emerg Med.

[11] Kaneki T, Kubo K, Kawashima A, Koizumi T, Sekiguchi M, Sone S (2000). Spontaneous pneumomediastinum in 33 patients: yield of chest computed tomography for the diagnosis of the mild type.. Respiration.

[12] Abrahamian FM, Pollack CV (2000). Traumatic pneumomediastinum caused by isolated blunt facial trauma: a case report.. J Emerg Med.

[13] Freixinet J, García F, Rodríguez PM, Santana NB, Quintero CO, Hussein M (2005). Spontaneous pneumomediastinum long-term follow-up.. Respir Med.

